# Toxicoproteomics Disclose Pesticides as Downregulators of TNF-α, IL-1β and Estrogen Receptor Pathways in Breast Cancer Women Chronically Exposed

**DOI:** 10.3389/fonc.2020.01698

**Published:** 2020-08-28

**Authors:** Luciana Pizzatti, Aedra Carla Bufalo Kawassaki, Bruna Fadel, Fabio C. S. Nogueira, Joseph A. M. Evaristo, Nicole Woldmar, Géssica Tuani Teixeira, Janaína Carla Da Silva, Thalita Basso Scandolara, Daniel Rech, Luciano Pessôa Zanetti Candiotto, Guilherme Ferreira Silveira, Wander Rogério Pavanelli, Carolina Panis

**Affiliations:** ^1^Federal University of Rio de Janeiro, Rio de Janeiro, Brazil; ^2^State University of West Paraná, UNIOESTE, Paraná, Brazil; ^3^Carlos Chagas Institute, Curitiba, Brazil; ^4^State University of Londrina, Londrina, Brazil

**Keywords:** breast cancer, toxicoproteomics, tumor necrosis factor alfa, interleukin beta 1, estrogen signaling

## Abstract

Deleterious effects have been widely associated with chronic pesticide exposure, including cancer development. In spite of several known consequences that pesticides can trigger in the human body, few is known regarding its impact on breast cancer women that are chronically exposed to such substances during agricultural work lifelong. In this context, the present study performed a high-throughput toxicoproteomic study in association with a bioinformatics-based design to explore new putative processes and pathways deregulated by chronic pesticide exposure in breast cancer patients. To reach this goal, we analyzed comparatively non-depleted plasma samples from exposed (*n* = 130) and non-occupationally exposed (*n* = 112) women diagnosed with breast cancer by using a label-free proteomic tool. The list of proteins differentially expressed was explored by bioinformatics and the main pathways and processes further investigated. The toxicoproteomic study revealed that women exposed to pesticides exhibited mainly downregulated events, linked to immune response, coagulation and estrogen-mediated events in relation to the unexposed ones. Further investigation shown that the identified deregulated processes and pathways correlated with significant distinct levels tumor necrosis factor alpha and interleukin 1 beta in the blood, and specific clinicopathological characteristics pointed out by bioinformatics analysis as adipose-trophic levels, menopause and intratumoral clots formation. Altogether, these findings reinforce pesticides as downregulators of several biological process and highlight that these compounds can be linked to poor prognosis in breast cancer.

## Introduction

Pesticides are chemical compounds widely used in agriculture to control pests since 1960s (1). Regardless of its specific targets, such substances unfortunately reach the human organism, and negative cumulative effects have been reported in people worldwide (2). In this context, chronic exposure to pesticides has been discussed as a significant risk factor for the development of cancer, including breast tumors (3).

Breast cancer is the most frequently malignant neoplasia diagnosed in women worldwide, whose origin is mostly connected to life habits and the environment, and to a lesser extent to inheritable genetic mechanisms (4). Therefore, the contribution of substances that are continuously present as contaminants in the environment, may have a pivotal role in the genesis of breast cancer (3), especially in geographic areas in which women are important players in the rural work and are in constant contact with these compounds (5, 6).

*In vitro* and experimental studies have reported the mechanisms triggered by pesticides that contribute to breast carcinogenesis, which fall essentially within DNA damage-based events in association with hormones deregulation and rising of metabolites that activate oncogenes (7, 8). However, few is known about how these mechanisms interconnects, as well as its correlation with the disease prognosis and clinicopathological features in human breast cancer as a result of the toxic consequences of pesticide exposure.

In the last years, aiming to expand the knowledge beyond isolated biological findings, high throughput molecular approaches combined with bioinformatics designs has raised as powerful tools to understand tumor behavior and biology. Thereby, it became possible to demonstrate that breast cancer is a challenging disease and have distinct mechanisms activated depending on specific clinicopathological characteristics (9–12).

As far as we know, until now there is no studies reporting the use of proteomics-based strategies to assess the clinical impact of chronic pesticide exposure in women with breast cancer. Relevant information, as how the deregulated biological processes induced by pesticides are interconnected to clinical parameters, are still missing. To fill this gap, the present study proposes a toxicoproteomic perspective to investigate the systemic profile of differentially expressed proteins in blood samples of breast cancer patients chronically exposed to pesticides. By using a high-throughput label-free proteomic strategy, we provide an integrative clinicopathological view based on deregulated events pointed out by bioinformatics approaches and corroborated by further analytical investigations.

## Materials and Methods

### Design of the Study, Patient Selection and Sample Collection

This is a prospective study conducted between May 2015 and December 2018 approved by the Institutional Ethics Board under the number CAAE 35524814.4.0000.0107. The sample size included 242 patients diagnosed with breast cancer attended by the 8th Health Care Region of the State of Paraná at Francisco Beltrão Cancer Hospital, Paraná, Brazil, which corresponds to a total of 27 municipalities. All patients signed consent forms and each protocol followed the principles for medical research involving human subjects from the Declaration of Helsinki. The Reporting Recommendations for Tumor Marker Prognostic Studies (REMARK) criteria were followed regarding patient selection, assay performance, and data analysis throughout the study.

To characterize the exposure, patients were invited to answer a questionnaire with 61 questions about their current and past occupational history. Based on their answers, we categorized the study population as occupationally exposed or not to pesticides. The criteria of inclusion applied in this study for the exposed group was chronic direct contact with pesticides (pesticide dilution and spraying without personal protection equipment – PPE and/or washing of contaminated clothes and PPE without gloves, at least once a month for the last 40 years). The main objective was to identify whether and how the patient had contact with pesticides: applying it directly or indirectly (by washing clothes or touching equipment and pesticide packs or merely living in areas where pesticides has been used). Unexposed group was composed mainly by women that lived most of their life in urban areas, without any occupational contact with pesticides. Therefore, patients were categorized according to their occupational status as exposed (*n* = 130) and non-occupationally exposed or unexposed (*n* = 112) to pesticides. Patients from both groups were matched for age.

Heparinized blood samples were collected by peripheral venous puncture (5 mL), centrifuged at 4000 rpm for 5 min and plasma aliquots kept frozen at −20°C until analysis. Clinical records were assessed to obtain clinicopathological information. Aiming to avoid any potential bias induced by chemotherapy, all patients included in the study were not under any treatment.

### Proteomic Analysis

#### In-Solution Tryptic Digestion

Proteomics analysis was performed on both groups using the pooled plasma samples of exposed and unexposed patients as strategies previously described (11, 12). Protein concentrations of cleared supernatants were determined using the Qubit™ Protein Assay Kit (Life Technologies). Samples were concentrated and ex-changed with 50 mM ammonium bicarbonate using a 3-kD ultra-filtration device (Millipore). Proteins extracts (50 μg) were then denatured (0.1% RapiGEST at 60°C for 15 min, Waters, Milford, United States), reduced (10 mM dithiothreitol at 60°C for 30 min), alkylated (10 mM iodoacetamide for 30 min at room temperature in the dark), and enzymatically digested with trypsin at a 1:50 (w/w) enzyme to protein ratio. Digestion was terminated by the addition of 10 μL of 5% trifluoroacetic acid (TFA) (13). Peptides were desalted in C18 micro columns (Harvard apparatus), dried in a vacuum centrifuge, resuspended in 0.1% formic acid, quantified by Qubit protein assay, and analyzed by label-free analysis.

#### LC-MS/MS Analyses

Two μg of digested peptides was analyzed in technical triplicate after 3 h of gradient (5% to 40% B/167 min; 40% to 95% B/5 min; and 95% B/8 min). Easy-nanoLC1000 (Thermo fisher) solvent A consisted of [95% H2O/5% acetonitrile (ACN)/0.1% formic acid] and solvent B of (95% ACN/5% H2O/0.1% formic acid). Trap-column used was Easy column C18, 2 cm × 100 μm i. d. × 5 μm, 120Å and analytical column of 25 cm and internal diameter of 75 μm (3 μm spheres, Reprosil Pur C18). Label-free quantification was performed in an Easy-nLC 1000 (Thermo Scientific) coupled to a QExactive Plus in FullScan-DDA MS2 mode used a dynamic exclusion list of 45 s and spray voltage at 2.70 kV. Full scan was acquired at a resolution of 70000 at m/z 200, with a m/z range of 350-2000, AGC of 1 × 106, and injection time of 50 ms. Selection of the 15 most intense ions for HCD fragmentation used a normalized collision energy of 30, precursor isolation window of m/z 1.2 and 0.5 offset, a resolution of 17 500 at m/z 200, AGC at 5 × 10^5^, and injection time of 100 ms (14).

#### Data Analysis

All samples replicate data were analyzed by the Proteome Discoverer 2.1 software using human database UniProt (V. Nov 2018-https://www.uniprot.org/). The parameters used were: full-tryptic search space, up to two missed cleavages allowed for trypsin, precursor mass tolerance of 10 ppm, and fragment mass tolerance of 0.05 Da. Carbamidomethylation of cysteine was included as fixed modification, and methionine oxidation and protein N-terminal acetylation were included as dynamic modifications in label-free quantification. Spectra analyses used a target-decoy strategy considering maximum delta CN of 0.05, all available peptide-spectrum matches, and a target false discovery rate (FDR) 0.01 (strict) as described previously (14). Parameters in the peptide filter were set up for high confidence with a minimum peptide length of six amino acids. For protein filter, we considered the minimum number of peptide sequence as 1, counting only rank 1 peptide. Peptides shared between multiple proteins was counted for the top scoring protein. The confidence thresholds in FDR protein validator were 0.01 for target FDR (strict). The strategy for protein grouping was strict parsimony. Statistical analysis was performed using Perseus Computational Platform v 1.6.10.50. The strategy with both datasets was Log2 transformation followed by subtract median normalization Student *t* test (*p*-value < 0.05). Volcano plot distribution presented demonstrated the log *p*-value vs log2 fold differences values. The process and biological pathway validation strategies was previously used and discussed by Pizzatti et al. (13) and Gjertsen and Wiig (15).

### *In silico* Functional Analysis

Functional *in silico* analysis were carried out with FunRich: Functional Enrichment Analysis Tool and MetaCore software using the integrated databases: Human Protein Reference Database (HPRD); Entrez Gene and Uniprot for biological pathways and biological process. For protein-protein interactions the following data bases were accessed: BioGRID; Intact e Human Proteinpedia. For localization, expression data and signaling pathways information the databases: Human Protein Altas; Human Proteome Browser; Human Proteome Map, Proteomics DB, Reactome; NCI; Cell map; and HumanCyc were used. The transcription factor consensus sequences prediction data were obtained with automated search in 29 mammals data bases on the fly in the FunRich tool followed by Bonferroni statistical analysis. Interaction network analysis was evaluated with String functional protein association network database^1^ using network analysis based on evidence with minimum required interaction score of 0.400. Venn Diagram the tool^2^ was used.

### Clinicopathological-Applied Investigation

Based on the results obtained from high-throughput proteomic screening and *in silico* data analysis, we investigated the main downregulated events triggered by chronic pesticide exposure in breast cancer patients individually.

Considering that most of the processes and pathways were downregulated by pesticide exposure, and that several of these events were connected with inflammation and immune response, we chose to measure two central cytokines commonly produced by patients in breast cancer (16, 17), the tumor necrosis factor alpha (TNF-α), and interleukin 1 beta (IL-1β). Analyses were performed by using commercial antibody-specific enzyme-linked immunosorbent assay (ELISA) kits (eBioscience Inc, United States) with internal controls. Results were calculated in pg/mL by fitting the data to a standard curve obtained using human recombinant cytokines.

Aiming to perform further investigation based on processes and pathways pointed out by bioinformatics, TNF-α and IL-1β results were categorized according to clinicopathological parameters of patients, which included age at diagnosis (cut-off at 50 years), tumor histological grade (I, II, and III), estrogen (ER), and progesterone (PR) receptors expression, lymph node metastasis presence or absence, intratumoral clots presence or absence, menopausal status at diagnosis, tumor size (≤2 cm, >2 cm, and <5 cm, ≥5 cm), ki-67 proliferation index (cut-off at 14%), and trophic-adipose levels based on body mass index (eutrophic ≤24.9 kg/m2, overweight between 25.0 and 29.9 kg/m2, and obese ≥ 30.0 kg/m2).

### Statistical Analysis

Experiments were carried out in duplicate datasets. Results were analyzed by using the Grubbs test for outlier detection and compared by Student’s *t*-test, Mann–Whitney or ANOVA, according to variances distribution and the number of the groups compared. *P*-value < 0.05 was considered significant. All statistical analyses were performed using GraphPad Prism version 7.0 (GraphPad Software, San Diego, CA, United States).

## Results

Detailed clinicopathological data of all patients included in the study are shown in Supplementary Table 1. Both groups presented similar ages at diagnosis and homogeneous distribution regarding other clinicopathological parameters. The mean age at diagnosis for unexposed patients was 55.2 years, ranging from 33 to 81 years, while the mean age at diagnosis for the occupationally exposed patients was 54.6 years, ranging from 30 to 86 years (please see the figure below). The comparison between groups retrieved a not significant *p* value of 0.7570 (Student’s *t*-test). No statistical differences were found when comparing each specific parameter between both groups.

Regarding the characterization of pesticide exposure, exposed women reported that they lived at least 50% of their lives working with pesticides, and to work at least once a week in direct contact with pesticides, as the following: 1. washing the clothes and personal protection equipment impregnated with pesticides worn by family members who applied these substances, 2. Preparing and diluting the concentrated pesticides, 3. Helping the spraying of diluted pesticides in the crops (time estimated: 4–8 h per day, during 2–3 consecutive days, each 1–2 weeks). Considering that Brazil has practically 100% of its arable land and that the area of study is one of the major regions of agriculture in Paraná State (second gross domestic product of the State), the occupational exposure of such patients is very intense. Further, 94% of interviewed women from exposed group reported to perform all activities without wearing personal protection equipment, not even gloves. Taking into consideration that pesticides are majorly absorbed by skin, and the duration/chronicity of the contact of patients with these substances, this is a worthwhile route of contamination, bigger than any other source as food or water. On the other hand, not occupationally exposed women did not exert any type of rural work enrolling pesticides, neither have any occupational contact with pesticides. When they answered the questionnaire, they reported none occupational contact (current or past) with pesticides. These women do not have the history of washing clothes or personal protection equipment contaminated with pesticides, and never had to apply pesticides in the crops. Therefore, they were selected to the unexposed group due to their completely distinct profile of occupational exposure history.

It is important to highlight that food consumption habits are similar in both exposed and unexposed groups, and the water system that supplies the study area is the same. Whether it is contaminated with pesticides, the occupational exposure overlaps it, due to its intensity and severity in the exposed group. Considering the intensity of chronic occupational exposure to pesticides, we considered that there is substantial information about pesticide exposure in the population of the study, and that the exposed patients are under completely distinct conditions if compared to the unexposed ones, independent on their contact with food and water pesticides (that are the same levels for both groups).

The proteomic screening identified in exposed and unexposed replicates a total of 554 proteins, with 280 proteins in exposed and 274 in unexposed ones. Information about total protein ratios and its raw data are reported in Supplementary Tables 2, 3.

**FIGURE 1:**
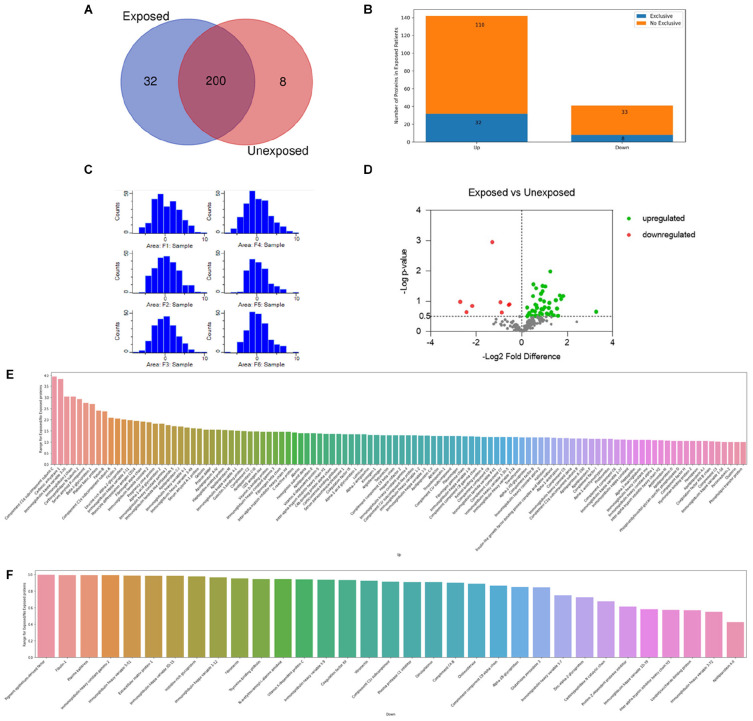
Proteouiic screening results. **(A)** Venn diagram showing the qualitative distribution of the identified proteins in both groups, 200 proteins identified in both conditions, 32 proteins identified in exclusively in exposed samples, and 8 proteins identified exclusively in unexposed samples. **(B)** The bars indicate the number of up and downregulated proteins differentially expressed in the exposed patients. Regarding the differentially expressed proteins when comparing the groups_:_ a total of 142 proteins were found upregulated and 41 downregulated in the exposed ones. **(C)** Histogram of distribution after normalization of the replicates. Exposed (Fl, F2, and F3) and unexposed (F4, F5, and F6). **(D)** Volcano plot of the differentially expressed proteins between exposed and unexposed samples. *In* green and red upregulated and downregulates proteins respectivelly. Tn gray proteins with -log *p*-value < 0.5. Total proteins identified as up **(E)** or downregulated **(F)** are reported accordingly to their specific ratios.

The Venn diagram (Figure 1A) shows the qualitative distribution of the identified proteins: 200 proteins identified in both conditions: 32 proteins identified in exclusively in exposed samples: and 8 proteins identified exclusively in unexposed samples. Regarding the differentially expressed proteins when comparing the groups, a total of 142 proteins were found upregulated and 41 downregulated in the exposed ones (Figure 1B).

The histogram distribution obtained after a Log2 transformation and subtract median normalization is shown in Figure 1C. General Label-free quantification analysis between the proteomic profiles identified 183 proteins differentially expressed. 142 proteins with exposed/unexposed ratio ≥ 1 and 41 proteins with exposed/unexposed ratio ≤ 1 (Supplementary Table 2 and Figures 1E,F). Between the initial 554 identified proteins in both groups, 243 protein presented quantified valid values and were present in three of three sample replicates in both groups. After the statistical analysis in Perseus software and appropriated filtering, 42 proteins were found upregulated and 8 proteins were found downregulated as shown in the volcano plot (Figure 1D). In Table 1, the list of differentially proteins showed in the volcano plot are presented with the respective Log *P*-values and Log2 fold differences.

**TABLE 1:** Differentially expressed proteins in chronically pesticide-exposed patients. Volcano plot data.

Accession (UniProt)	Description	−Log2 fold difference	−Log *p*-value
**Down regulated proteins in exposed samples**			
Q15848	Adiponectin OS = Homo sapiens OX = 9606 GN = ADIPOQ PE = 1 SV = 1	−0,577652	0,877936
P02652	Apolipoprotein A-II OS = Homo sapiens OX = 9606 GN = APOA2 PE = 1 SV = 1	−1,29743	2,95751
P15169	Carboxypeptidase N catalytic chain OS = Homo sapiens OX = 9606 GN = CPN1 PE = 1 SV = 1	−0,5372	0,903306
P12259	Coagulation factor V OS = Homo sapiens OX = 9606 GN = F5 PE = 1 SV = 4	−2,18176	0,848073
A0A0B4J1Y9	Immunoglobulin heavy variable 3-72 OS = Homo sapiens OX = 9606 GN = IGHV3-72 PE = 3 SV = 1	−0,937188	0,972346
A0A0A0MT36	Immunoglobulin kappa variable 6D-21 OS = Homo sapiens OX = 9606 GN = IGKV6D-21 PE = 3 SV = 1	−2,69985	0,985784
P01721	Immunoglobulin lambda variable 6-57 OS = Homo sapiens OX = 9606 GN = IGLV6-57 PE = 1 SV = 2	−2,4305	0,645198
P18428	Lipopolysaccharide-binding protein OS = Homo sapiens OX = 9606 GN = LBP PE = 1 SV = 3	−0,877242	0,632545
**Up regulated proteins in exposed samples**			
P02763	Alpha-1-acid glycoprotein 1 OS = Homo sapiens OX = 9606 GN = ORM1 PE = 1 SV = 1	0,282142	0,549393
P19652	Alpha-1-acid glycoprotein 2 OS = Homo sapiens OX = 9606 GN = ORM2 PE = 1 SV = 2	0,672468	0,780538
P01019	Angiotensinogen OS = Homo sapiens OX = 9606 GN = AGT PE = 1 SV = 1	0,598796	0,51995
P06727	Apolipoprotein A-IV OS = Homo sapiens OX = 9606 GN = APOA4 PE = 1 SV = 3	0,811802	0,619374
P02655	Apolipoprotein C-II OS = Homo sapiens OX = 9606 GN = APOC2 PE = 1 SV = 1	0,237511	0,792502
P02749	Beta-2-glycoprotein 1 OS = Homo sapiens OX = 9606 GN = APOH PE = 1 SV = 3	1,20994	0,951809
P04003	C4b-binding protein alpha chain OS = Homo sapiens OX = 9606 GN = C4BPA PE = 1 SV = 2	0,446286	0,52209
P00915	Carbonic anhydrase 1 OS = Homo sapiens OX = 9606 GN = CA1 PE = 1 SV = 2	1,67218	1,19446
P00918	Carbonic anhydrase 2 OS = Homo sapiens OX = 9606 GN = CA2 PE = 1 SV = 2	0,632465	1,43293
P22792	Carboxypeptidase N subunit 2 OS = Homo sapiens OX = 9606 GN = CPN2 PE = 1 SV = 3	1,25728	1,98658
P04040	Catalase OS = Homo sapiens OX = 9606 GN = CAT PE = 1 SV = 3	0,913062	1,51147
O43866	CD5 antigen-like OS = Homo sapiens OX = 9606 GN = CD5L PE = 1 SV = 1	0,583446	0,511082
P02745	Complement C1q subcomponent subunit A OS = Homo sapiens OX = 9606 GN = C1QA PE = 1 SV = 2	0,816096	1,05262
P02747	Complement C1q subcomponent subunit C OS = Homo sapiens OX = 9606 GN = C1QC PE = 1 SV = 3	3,27164	0,659602
P0C0L4	Complement C4-A OS = Homo sapiens OX = 9606 GN = C4A PE = 1 SV = 2	1,03893	1,49116
P07360	Complement component C8 gamma chain OS = Homo sapiens OX = 9606 GN = C8G PE = 1 SV = 3	0,24517	0,503356
Q9UGM5	Fetuin-B OS = Homo sapiens OX = 9606 GN = FETUB PE = 1 SV = 2	1,40429	0,541632
P02671	Fibrinogen alpha chain OS = Homo sapiens OX = 9606 GN = FGA PE = 1 SV = 2	1,10486	0,59767
O75636	Ficolin-3 OS = Homo sapiens OX = 9606 GN = FCN3 PE = 1 SV = 2	1,00807	0,802906
Q08380	Galectin-3-binding protein OS = Homo sapiens OX = 9606 GN = LGALS3BP PE = 1 SV = 1	0,465231	0,6933
P06396	Gelsolin OS = Homo sapiens OX = 9606 GN = GSN PE = 1 SV = 1	0,511214	1,56289
P00739	Haptoglobin-related protein OS = Homo sapiens OX = 9606 GN = HPR PE = 2 SV = 2	1,34752	0,539945
P01857	Immunoglobulin heavy constant gamma 1 OS = Homo sapiens OX = 9606 GN = IGHG1 PE = 1 SV = 1	0,585063	0,669012
P01742	Immunoglobulin heavy variable 1-69 OS = Homo sapiens OX = 9606 GN = IGHV1-69 PE = 1 SV = 2	0,64299	0,872349
A0A0B4J1V2	Immunoglobulin heavy variable 2-26 OS = Homo sapiens OX = 9606 GN = IGHV2-26 PE = 3 SV = 1	0,33751	0,617746
P04433	Immunoglobulin kappa variable 3-11 OS = Homo sapiens OX = 9606 GN = IGKV3-11 PE = 1 SV = 1	1,30189	0,644815
P01619	Immunoglobulin kappa variable 3-20 OS = Homo sapiens OX = 9606 GN = IGKV3-20 PE = 1 SV = 2	1,54984	0,759295
A0A0C4DH55	Immunoglobulin kappa variable 3D-7 OS = Homo sapiens OX = 9606 GN = IGKV3D-7 PE = 3 SV = 5	1,82442	1,17608
P06312	Immunoglobulin kappa variable 4-1 OS = Homo sapiens OX = 9606 GN = IGKV4-1 PE = 1 SV = 1	0,870637	0,741953
P0DOY2	Immunoglobulin lambda constant 2 OS = Homo sapiens OX = 9606 GN = IGLC2 PE = 1 SV = 1	0,73101	0,509415
P01703	Immunoglobulin lambda variable 1-40 OS = Homo sapiens OX = 9606 GN = IGLV1-40 PE = 1 SV = 2	0,980606	0,997284
P01700	Immunoglobulin lambda variable 1-47 OS = Homo sapiens OX = 9606 GN = IGLV1-47 PE = 1 SV = 2	1,59502	0,521646
P01701	Immunoglobulin lambda variable 1-51 OS = Homo sapiens OX = 9606 GN = IGLV1-51 PE = 1 SV = 2	0,495094	1,1716
Q14624	Inter-alpha-trypsin inhibitor heavy chain H4 OS = Homo sapiens OX = 9606 GN = ITIH4 PE = 1 SV = 4	0,622172	0,533977
P02750	Leucine-rich alpha-2-glycoprotein OS = Homo sapiens OX = 9606 GN = LRG1 PE = 1 SV = 2	0,941312	0,739914
P08571	Monocyte differentiation antigen CD14 OS = Homo sapiens OX = 9606 GN = CD14 PE = 1 SV = 2	1,18636	0,777581
P02776	Platelet factor 4 OS = Homo sapiens OX = 9606 GN = PF4 PE = 1 SV = 2	0,896119	0,575848
P20742	Pregnancy zone protein OS = Homo sapiens OX = 9606 GN = PZP PE = 1 SV = 4	0,918109	1,33271
P02760	Protein AMBP OS = Homo sapiens OX = 9606 GN = AMBP PE = 1 SV = 1	1,17521	0,568105
Q92954	Proteoglycan 4 OS = Homo sapiens OX = 9606 GN = PRG4 PE = 1 SV = 3	0,841435	1,25299
P0DJI8	Serum amyloid A-1 protein OS = Homo sapiens OX = 9606 GN = SAA1 PE = 1 SV = 1	0,591495	0,891718
P02743	Serum amyloid P-component OS = Homo sapiens OX = 9606 GN = APCS PE = 1 SV = 2	1,50171	1,03638
P62979	Ubiquitin-40S ribosomal protein S27a OS = Homo sapiens OX = 9606 GN = RPS27A PE = 1 SV = 2	1,73161	1,07562

**FIGURE 2:**
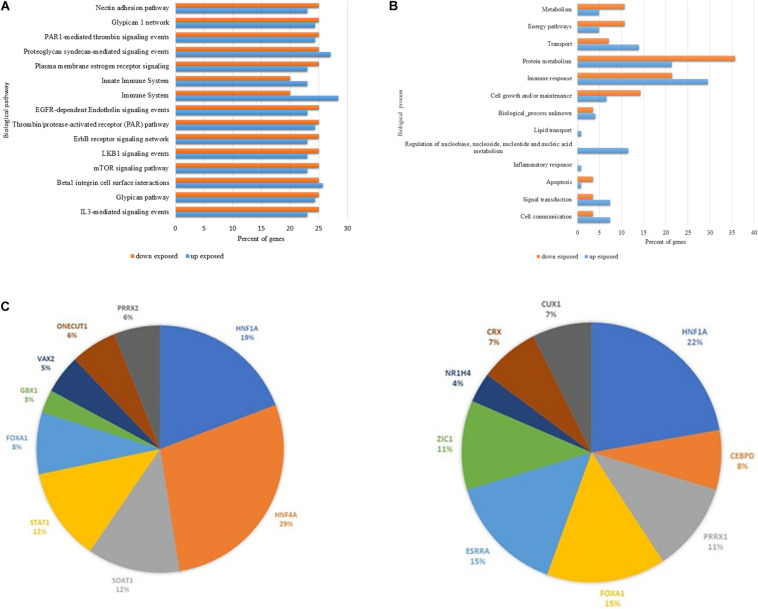
*In stiico* comparative analysis between the main up and down regulated proteins differentially expressed by breast cancer patients chronically exposed to pesticides. **(A)** Biological pathways. **(B)** Biological processes and **(C)** Prediction of potential Transcription fator related to proteins data set regulation. Left panel prediction for up regulated proterins. Right panel prediction for downregulated proteins. The orange columns represent the downregulated events, and the blue columns indicate the upregulated ones. Note that for most of the events, the downregulation is more frequent.

The *in silico* analysis (Figure 2) performed in FunRich: Functional Enrichment Analysis Tool software revealed the major biological events in which all the up and downregulated proteins differently expressed in the chronically pesticide-exposed patients are enrolled. Figure 2A shows an amount of 15 biological pathways in which 11 are found downregulated in the chronically pesticide-exposed patients. These pathways were related to adhesion (5), receptors and second messengers signaling (5), immune response (3), and coagulation mechanisms (2). Most of them are intrinsically connected with immune-mediated events. In respect to the 13 biological processes identified through the bioinformatics evaluation (Figure 2B), 5 were predominantly downregulated in the chronically pesticide-exposed group of patients. The processes affected were related to metabolism and energy, protein metabolism, cell growth, and apoptosis.

Using the Funrich data base for transcription factors consensus binding sites sequences, the prediction of potential transcriptional factors related (direct or indirectly) with the identified protein data sets, were predicted. In Figure 2C is showed the top nine transcription factor (DNA consensus sequences) that are present in the promoter sequences of the identified proteins. It is noted that most of the proteins were related to Hepatocyte nuclear factor 1-alpha HNF1A (in downregulated dataset), HNF4A Hepatocyte nuclear factor 4-alpha (in upregulated data set), Estrogen-related receptor alpha (ESRRA; in downregulated data set) Forkhead box protein A1 FOXA1 (in downregulated data set) and Signal transducer and activator of transcription 1-alpha STAT1 (upregulated data set).

**FIGURE 3:**
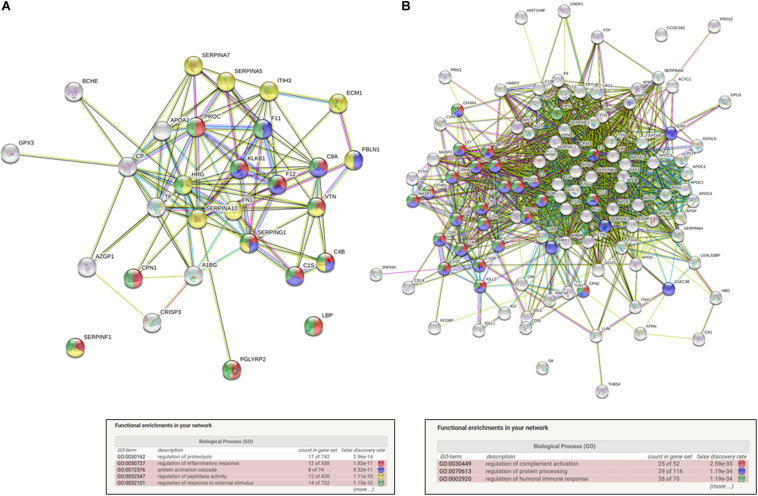
*In silico* functional interaction enrichement network analysis of differentially expressed proteins in breast cancer patients chronically exposed to pesticides. **(A)** Downregulated dataset interaction networks, number of nodes: 25; number of edges: 75; average node degree: 6; avg. local clustering coefficient: 0.605; and expected number of edges: 3: PPI enrichment *p*-value: <1.0e-16. **(B)** Up regulated dataset interaction networks. Number of nodes: 32 number of edges; 109 average node degree; 6.81 avg. local; clustering coefficient: 0.611; expected number of edges: 12; and PPI enrichment p-value: <10e-16.

Moreover, the functional protein association networks were analyzed. In String data base software, using network analysis based on evidence with minimum required interaction score of 0.400 using its database. The list of proteins used to perform this analysis is shown in Supplementary Table 2 and in Table 1. The results of the network analysis retrieved from String database software is shown in Supplementary Tables 4, 5. The functional enrichments interactions observed after the *in silico* analysis of the up and down regulated datasets showed an enrichment *p*-value of < 1.0e-16 and are presented in the Figure 3. This means that proteins presented in the identified data set have more interactions among themselves than what would be expected for a random set of proteins of similar size, drawn from the genome. Such an enrichment indicates that the proteins are at least partially biologically connected, as a group. For the downregulated protein dataset (Figure 3A), this *in silico* analysis also showed as top 5 Biological process (GO) hits: regulation of inflammatory response (false discovery rate of 1.82e-11); protein activation cascade (false discovery rate 9.32e-11); regulation of peptidase activity (false discovery rate 1.11e-10); and regulation of response to external stimulus (false discovery rate 1.15e-10) as major biological process interaction. The major biological process identified in the network interaction of the upregulated identified dataset (Figure 3B) are regulation of complement activation (false discovery rate of 2.59e-35); regulation of protein processing (false discovery rate 1.19e-34) and regulation of humoral immune response (false discovery rate 1.19e-34).

According to the results from the *in silico* study we performed a search in the literature to understand the putative connections among the highlighted processes and pathways downregulated in breast cancer patients by chronic pesticide exposure. Based on this, it was possible to note that most of data were linked to inflammation and immune-related events. Therefore, aiming to understand the clinicopathological meaning of our findings and validate the biological relevance and data reliability, we decided to investigate TNF-α and IL-1β levels in plasma samples from both exposed and unexposed groups. TNF-α and IL-1β represents major key factor of inflammation and immune-related events and process and pathway validation strategies, as also previously described (13, 18). These cytokines were chosen due to its well-known production in breast cancer patients (16, 17). To validate the downregulated biological events, these cytokines levels were analyzed considering both general levels and some clinicopathological parameters related to the particular processes and pathways revealed by the *in silico* study. Cytokine levels distributed according to the investigated clinicopathological parameters are reported in Supplementary Tables 6, 7.

**FIGURE 4:**
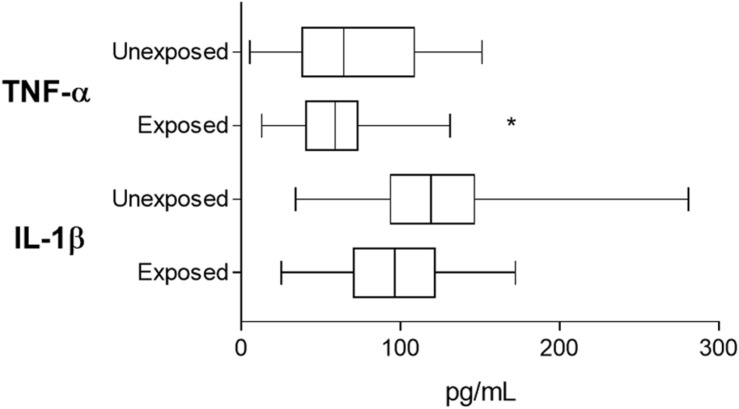
Plasmatic TNF-α and IL-lβ measurements for validation of imune response related events as pointed out by proteomic screening. Based on the results obtained from proteomic screening that shown the downregulation of imune response and inflammation-related events, the validation step investigated two major cytokines reported as produced in breast cancer patients. *indicates statistical difference (*p* < 0.05). Student’s *t* Test.

Tumor necrosis factor alpha levels were significantly reduced in the chronically pesticide-exposed patients (Figure 4) when compared to those unexposed (94.31 ± 6.02 pg/mL for exposed and 122.6 ± 10.78 pg/mL for unexposed, *p* = 0.0378). IL-1β levels did not differ (59.24 ± 4 pg/mL for exposed and 71.1 ± 7.62 pg/mL for unexposed, *p* = 0.3067).

**FIGURE 5:**
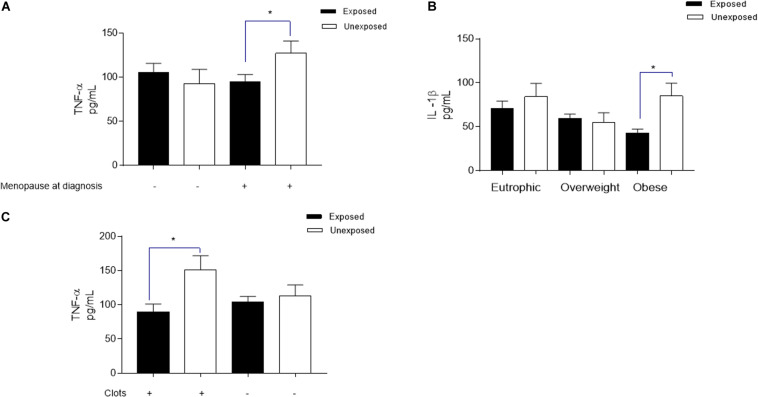
Significant variations in plasmatic TNF-α and IL-lβ levels from breast cancer patients exposed or unexposed to pesticides according to specific clinicopathological profiles. Clinicopathological parameters investigated were chosen based on the downregulated results obtained from biological pathways, biological processes, and molecular functions revealed by *in silica* analysis in breast cancer cancer chronically exposed to pesticides. For all parameters_:_ the levels of both cytokines were investigated; and here are represented only those with statistical significance for each cytokine. **(A)** For investigation of the plasma membrane estrogen receptor signaling and the transcription factor estrogen-related receptor alpha (ESRRA) pointed out by *in silico* analysis as downregulated (identified in the biological pathways and TFs analysis), the measurement of TNF-ct levels distributed according to patients menopausal status at diagnosis. **(B)** Regarding the metabolism and energy pathways downregulation (identified in the biological processes analysis), the measurement of EL-lβ levels in plasma of breast cancer patients according to their trophic-adipose status, categorized by ther body mass index as Eutrophic, Overweight and obese. **(C)** To validate the downregulated PAR-1-mediated thrombin signaling events (identified in the biological pathways analysis), TNF-α levels were categorized according to the formation of intratumoral clots observed during biopsies microscopic analysis. *indicates statistical difference (*p* < 0.05).

To explore the plasma membrane estrogen receptor signaling downregulation, as well the involvement of the downregulated transcription factor ESRRA pointed out by *in silico* analysis, cytokine levels were analyzed according to patients’ menopausal status at diagnosis. TNF-α levels (Figure 5A) was significantly reduced in breast cancer patients that are chronically exposed to pesticides when compared to the unexposed ones (94.6 ± 8.52 pg/mL and 127.2 ± 13.99 pg/mL, respectively, *p* = 0.0443), while IL-1β was not different.

Concerning the study of metabolism and energy pathways downregulation (Figure 5B) suggested by *in silico* analysis, IL-1β levels revealed significantly reduced in the plasma of obese breast cancer patients chronically exposed to pesticides (42.78 ± 4.64 ρg/mL to the exposed and 85.10 ± 14.52 ρg/mL to the unexposed, *p* = 0.0247). For this pathway, TNF-α levels did not change. In relation to the validation of the downregulated PAR-1-mediated thrombin signaling, a clot formation-related pathway also highlighted in the *in silico* processes analysis, TNF-α levels were significantly reduced in chronically pesticide-exposed patients that presented intratumoral clots (Figure 5C, 89.82 ± 11.31 pg/mL for exposed patients and 151.0 ± 20.99 pg/mL for non-exposed patients, *p* = 0.0128). In this case, IL-1β has had no variation.

## Discussion

Toxicoproteomics is the field of proteomics that aims to understand the impact of environmental exposures on proteins, processes and pathways in biological systems by using global protein expression approaches (19). Based on this concept, the present study was designed to comprehend the influence of chronic pesticide exposure in systemic proteomic profile of breast cancer patients. Our results demonstrated that these patients have systemically deregulated processes in relation to the unexposed ones, pointing out pesticide exposure as significant downregulators of several biological networks.

Taking into account that such pathways are substantially linked to inflammatory and immune responses, we focused our validation analyses on the clinical impact of pesticide chronic exposure in breast cancer patients regarding their systemic cytokines profile. Furthermore, we pinpointed this scenario based on a set of prognostic parameters that correlates with the data supported by the toxicoproteomic-based results.

Cytokine production is a central mechanism triggered by immune response against cancer (20), and high levels of systemic TNF-α and IL-1β has been reported in breast cancer patients (16, 17). Our findings demonstrated that chronic exposure to pesticides impairs the systemic cytokine production in breast cancer patients, which leads to lower levels of both TNF-α and IL-1β under specific clinicopathological conditions, when compared to the unexposed group. Cytokine shutdown may result in immunosuppression, a process already documented as a consequence of chronic pesticide exposure (21) that may favor not only cancer development but also enhance its aggressiveness (22). Several pesticides are proven to be immunotoxicants, and a variety of deleterious mechanisms can be listed, including reduction in the number and function of immune cells (23), genetic damage in lymphocytes (24) and suppression of Th1 responses (25). TNF-α can control the activity of HNF1 (26), which helps to understand why it was referred as the main transcription factor downregulated by chronic pesticide exposure in our study.

Nevertheless, pesticides are known for their capability to cause endocrine disruption, which seems to contribute substantially for breast cancer development (27). We found that the most clinically relevant biological pathway pinpointed as downregulated by pesticide exposure in breast cancer patients by *in silico* was the estrogen receptor (ER) signaling, in association with the ESSRA transcription factor. The knockdown of ER axis in breast cancer patients could contribute to the development of the most aggressive phenotype of breast cancer, the triple negative (TNBC). The underlying biological mechanisms that drive TNBC development remains unclear, but ER loss seems to have a role in the metastatic processes. Studies have reported divergences between primary tumors and its metastases, highlighting that the loss of ER is a common event in breast cancer (28, 29), turning favorable prognosis luminal tumors into poor prognosis TNBC. Since pesticide exposure is a common event worldwide, it could be investigated as a possible mechanism in TBNC genesis. It is worth to mention that in this study there are TNBC patients in the exposed group in comparison to the unexposed one, reinforcing these findings.

Since ER signaling was downregulated in patients by pesticide exposure, we have advanced our investigation to determine if cytokine levels were differentially distributed in exposed patients according to their hormonal status. Pesticide exposure did not promote any significant change when comparing the non-menopausal group of patients. However, exposed patients presented significantly reduced TNF-α levels when compared to the unexposed, suggesting that the exposure may affects TNF-α production in the absence of estrogen. It is known that estrogen modulates Th1/Th17 immune responses (30), and can control the secretion of TNF-α by macrophages (31). Moreover, it is expected that during menopause an increase in TNF-α production occurs as a response to estrogen deprivation, as well as a compensatory mechanism against the decreasing of immune cells (32). However, we did not observe any of these situations in pesticide-exposed patients. Thus, we may conclude that disruptions in TNF-α antitumor mechanisms could result in disease aggravation for patients under pesticide exposure in the future. In this context, we identified that 3.3% of the patients from the unexposed group had recurrence of breast cancer, while about 19% of the occupationally exposed patients recurred. Therefore, a follow-up study is necessary to understand whether recurrence is related to cytokine levels.

Bringing together the results of metabolism and energy processes, both downregulated by *in silico* analysis, IL-1β levels was found significantly reduced in obese patients exposed to pesticides in comparison to the unexposed obese patients. Pesticides affects macrophages function by reducing their lysosomal activity, promoting negative regulation of IL-1β secretion. Additionally, pesticides appear to modulate IL-1β levels in the spleen and thymus of mice fed with high-fat diet (33), and promote its spontaneous secretion by human blood monocytes blood (34), which could lead into the exhaustion of this system. Considering that obesity constitutes a major risk factor for the worst prognosis of breast cancer (35), the reduction of IL-1β in these patients induced by pesticides could constitute an additional aggravating factor.

Among this context of inflammation mechanisms deregulated due to pesticides exposure, we also identified downregulation of components from the coagulation pathway. We observed significantly reduced TNF-α levels in breast cancer patients that exhibited intratumoral clots and were exposed to pesticides. This finding suggests an interplay between TNF-α and HNF1 induced by pesticides in breast cancer patients, since HNF1 directly controls the transcription of blood clotting genes (36) and both were downregulated accordingly to the *in silico* analysis.

In conclusion, the validation experiments performed by crossing cytokines levels and specific clinicopathological parameters, that were chosen based on the bioinformatics analysis, reinforced that toxicoproteomics is a reliable approach to investigate the impact of chronic pesticide exposure in breast cancer patients. These findings also support pesticides as critical downregulators of biological responses and mechanisms that can be implicated in breast cancer worse development and progression.

## Data Availability Statement

The mass spectrometry proteomics data have been deposited to the ProteomeXchange Consortium via the PRIDE [1] partner repository with the dataset identifier PXD018577.

## Ethics Statement

The studies involving human participants were reviewed and approved by State University of West Paraná IRB. The patients/participants provided their written informed consent to participate in this study.

## Author Contributions

LP, BF, FN, JE, and NW: proteomic and bioinformatic analysis, manuscript discussion, and writing. AK, JD, and TS: data analysis, cytokines measurement, clinicopathological data assessment, manuscript discussion, and writing. DR: patients selection, manuscript discussion, and writing. LC: pesticide exposure categorization, manuscript discussion, and writing. GS: bioinformatics, manuscript discussion, and writing. WP and CP: design of the study, data analysis, study supervision, manuscript discussion, and writing. All authors contributed to the article and approved the submitted version.

## Conflict of Interest

The authors declare that the research was conducted in the absence of any commercial or financial relationships that could be construed as a potential conflict of interest.

## Footnotes

1 https://string-db.org/

2 http://bioinformatics.psb.ugent.be/webtools/Venn/
