# Supplementary material for: Toxicoproteomics Disclose Pesticides as Downregulators of TNF-α, IL-1β and Estrogen Receptor Pathways in Breast Cancer Women Chronically Exposed
Source: Front Oncol. 2020 Aug 28;10:1698. doi: 10.3389/fonc.2020.01698 (PMC7483484; doi:10.3389/fonc.2020.01698)
Supplement: Supplementary file 1 [file Table_1.docx]

**Supplementary Table 1–** Clinicopathological data of breast cancer patients chronically exposed or not to pesticides enrolled in the study.

|  | **Exposed** | **Unexposed** |
| --- | --- | --- |
| **Age at diagnosis** |  |  |
| ≤ 50 years | 45.2% | 41.1% |
| > 50 years | 54.8% | 58.9% |
| **Histological grade** | |  |
| Grade I | 27.5% | 49.1% |
| Grade II | 47.5% | 30.9% |
| Grade III | 25.0% | 20.0% |
| **ER/PR expression** | |  |
| ER/PR positive + ki67<14% | 24.3% | 25.0% |
| ER/PR positive + ki67>14% | 27.0% | 50.0% |
| ER/PR negative and any ki67% | 37.8% | 25.0% |
| **Lymphnodal metastasis** | |  |
| No | 69.4% | 68.2% |
| Yes | 30.6%) | 31.8% |
| **Intratumoral clots** |  |  |
| No | 68.4%) | 75.0% |
| Yes | 31.6% | 25.0% |
| **Menopause at diagnosis** |  |  |
| No | 36.6% | 26.7% |
| Yes | 63.4% | 73.3% |
| **Tumor size** |  |  |
| ≤ 2cm | 40.6% | 38.5% |
| Between 2cm and 5cm | 25.0% | 53.8% |
| ≥ 5cm | 34.4% | 7.7% |
| **Ki-67** |  |  |
| < 14% | 30.0% | 35.5% |
| ≥ 14% | 60.0% | 64.5% |
| **Body mass index (kg/m^2^)** |  |  |
| Eutrophic | 31.0% | 33.3% |
| Overweight | 47.6% | 40.0% |
| Obese | 21.4% | 26.7% |

* Data are expressed as mean±standard errors of the means for parametric data and median (min-max) for no-parametric data. ER = estrogen receptors, PR = progesterone receptors. For all parameters reported here, no statistical differences were found when comparing each specific parameter between both groups (p>0.05).
