# Supplementary material for: Toxicoproteomics Disclose Pesticides as Downregulators of TNF-α, IL-1β and Estrogen Receptor Pathways in Breast Cancer Women Chronically Exposed
Source: Front Oncol. 2020 Aug 28;10:1698. doi: 10.3389/fonc.2020.01698 (PMC7483484; doi:10.3389/fonc.2020.01698)
Supplement: Supplementary file 6 [file Table_6.docx]

**Supplementary Table 6** – Circulating levels of TNF-α in breast cancer patients chronically exposed or not to pesticides according to their clinicopathological characteristics.

|  | **TNF-α (pg/mL)** | |  |
| --- | --- | --- | --- |
|  | **Exposed** | **Unexposed** | p value |
| **Age at diagnosis** |  |  |  |
| ≤ 50 years | 98.14±9.23 | 94.04 ±13.50 | 0.7972 |
| > 50 years | 96.50 ±9.25 | 123.90 ±12.61 | 0.0808 |
| **Histological grade** |  |  |  |
| Grade I | 114.40 ±13.00 | 118.80 ±15.67 | 0.8347 |
| Grade II | 112.90 ±17.88 | 157.90 ±36.32 | 0.2526 |
| Grade III | 100.70 ±10.95 | 110.90 ±21.06 | 0.6530 |
| **ER/PR expression** |  |  |  |
| ER/PR positive + ki67<14% | 97.90 (47.15-138.60) | 146.40(77.90-245.00) | 0.1416 |
| ER/PR positive + ki67>14% | 110.70 (75.03-121.80) | 109.30(93.95-133.60) | 0.8357 |
| ER/PR negative and any ki67% | 91.14±8.51 | 90.25±20.03 | 0.9615 |
| **Lymphnodal metastasis** |  |  |  |
| No | 104,30 (79.33-130.70) | 121.50 (86.05-177.20) | 0.1778 |
| Yes | 93.76 ±2.24 | 97.22±16.12 | 0.8646 |
| **Tumor size** |  |  |  |
| ≤ 2cm | 101.80 ±9,03 | 104.20 ±14.09 | 0.8810 |
| Between 2cm and 5cm | 87.29±13.45 | 111.90 ±15.42 | 0.2663 |
| ≥ 5cm | 111.00±16.24 | 121.50 ±2.15 | 0.7359 |
| **Ki-67** |  |  |  |
| < 14% | 86.73±10.46 | 116.60±20.59 | 0.1649 |
| ≥ 14% | 125.90±13.28 | 111.00 ±10.63 | 0.4061 |
| **Body mass index (kg/m^2^)** |  |  |  |
| Eutrophic | 93.60 (67.88-119.30) | 136.40 (92.90-224.30) | 0.0848 |
| Overweight | 108.60 ±10.25 | 121.20±18.50 | 0.5247 |
| Obese | 78.78 ±10.49 | 98.31±16.26 | 0,3190 |

* Data are expressed as mean±standard errors of the means for parametric data and median (min-max) for no-parametric data. ER = estrogen receptors, PR = progesterone receptors.
