# Supplementary material for: Toxicoproteomics Disclose Pesticides as Downregulators of TNF-α, IL-1β and Estrogen Receptor Pathways in Breast Cancer Women Chronically Exposed
Source: Front Oncol. 2020 Aug 28;10:1698. doi: 10.3389/fonc.2020.01698 (PMC7483484; doi:10.3389/fonc.2020.01698)
Supplement: Supplementary file 7 [file Table_7.docx]

**Supplementary Table 7** – Circulating levels of IL-1β in breast cancer patients chronically exposed or not to pesticides according to their clinicopathological characteristics.

|  | **IL-1β (pg/mL)** | |  |
| --- | --- | --- | --- |
|  | **Exposed** | **Unexposed** | p value |
| **Age at diagnosis** |  |  |  |
| ≤ 50 years | 57.30 (41.90-66.50) | 63.50 (38.10-108.80) | 0.4257 |
| > 50 years | 62.75 ±6.55 | 72.94 ±11.00 | 0.4007 |
| **Histological grade** |  |  |  |
| Grade I | 64.80 ±6.71 | 77.43 ±10.57 | 0.3445 |
| Grade II | 60.87 ±6.63 | 62.84 ±15.53 | 0.8913 |
| Grade III | 54.78 ±7.47 | 69.96 ±16.35 | 0.3642 |
| **ER/PR expression** |  |  |  |
| ER/PR positive + ki67<14% | 51.76±9.84 | 78.09±18.79 | 0.2067 |
| ER/PR positive + ki67>14% | 60.40(48.05-74.65) | 63.50(38.45-110.00) | 1.000 |
| ER/PR negative, any ki 67% | 56.82±6.37 | 53.83±10.36 | 0.8036 |
| **Lymphnodal metastasis** |  |  |  |
| No | 52.55±7.76 | 80.67±15.63 | 0.8293 |
| Yes | 61.35±5.70 | 58.88±11.38 | 0.0895 |
| **Intratumoral clots** |  |  |  |
| No | 57.30(36.73-69.05) | 64.20(36.55-108.80) | 0.6013 |
| Yes | 59.18±6.75 | 72.45±15.31 | 0.3695 |
| **Menopause at diagnosis** |  |  |  |
| No | 55.01±5.03 | 64.34±12.27 | 0.4079 |
| Yes | 61.55(37.10-79.05) | 64.20(36.55-100.00) | 0.4559 |
| **Tumor size** |  |  |  |
| ≤ 2cm | 47.37 ±5.82 | 49.29 ±5.37 | 0.8280 |
| Between 2cm and 5cm | 57.58±6.41 | 72.20±10.46 | 0.2773 |
| ≥ 5cm | 61.75±9.85 | 29.60 ±24.60 | 0.1960 |
| **Ki-67** |  |  |  |
| < 14% | 55.00(35.98-65.93) | 59.60(36.93-103.30) | 0.3162 |
| ≥ 14% | 67.61±5.47 | 71.39 ±9.48 | 0.7181 |

* Data are expressed as mean±standard errors of the means for parametric data and median (min-max) for no-parametric data. ER = estrogen receptors, PR = progesterone receptors.
